# Nursing Progress and Its Developments: A Year's Work at the College of Nursing

**Published:** 1918-06-15

**Authors:** 


### THE MATRONS' AND SISTERS' DEPARTMENT.

### NURSING PROGRESS AND ITS DEVELOPMENTS.

### A YEAR'S WORK AT THE COLLEGE OF NURSING.

Apart from the social aspects of annual meetings they are indispensable in all societies to the growth of a corporate sense. They are an occasion for looking back and for looking forward, for taking note of what has been accomplished and forecasting the policy to be adopted for the future. In both respects, in performance and in promise, the members of the College of Nursing have good reason to be satisfied with their College meeting.

For a budding society the main symptoms of healthy growth are to be sought in numbers and in finance. In both these directions Sir Arthur Stanley was able to produce really striking figures. In little over a year the College has enlarged its membership from 2,553 to 8,922. All initial expense of rent, salaries, postages, and so forth have been defrayed, and the accumulated fund shows that the entire fees paid by the nurses for registration, plus £300 to the good, have been invested. There is no compulsory yearly subscription to the College of Nursing. The guinea registration fee confers life membership. But members who can afford to do so are invited to send a yearly sum as a free contribution, and we confess we greatly prefer this spontaneous method to the tiresome and thankless one of collecting small sums from many thousands of workers who are constantly changing their addresses, getting married, and in other ways passing on to a different sphere.

Numbers and finance being in a particularly healthy condition, we must next inquire "What has the infant College done during the past year to justify its existence?" Last year it was well on the way towards amalgamation with the older incorporated Nurses' Association. The history of the year, so far as that is concerned, is that the project has fallen through. Sir Arthur Stanley told his hearers that many members of the College had consistently opposed the proposed amalgamation, and that the unprecedented increase in numbers already referred to had set in after the decision against amalgamation had been announced. There is nothing to regret, then, in this direction, and this much is gained—that the project is out of the way and no longer bars the road to complete self-government on the part of the College members. They have taken the first step towards a truly representative Council, and the twelve newly elected members of the Council have a direct mandate from nurses. In the matter of State Registration considerable progress has been made. An agreed Bill is actually now in the press and will shortly be in the hands of nurses for study and discussion. Lastly, the College during the past year has made rapid strides in the direction of decentralisation. Local centres have been already formed in nine towns, and are in course of formation in many more. An entirely new current of self-governing enthusiasm has begun to make itself felt among nurses through the length and breadth of the land.

It is possible, but not probable, that 1918-19 may bring with it the legislation on behalf of nurses so earnestly desired by many. The road to it lies through an irresistible appeal from nurses, joining the College in thousands upon thousands and speaking with one voice. No Government will deny or delay to give nurses at this time what they unanimously demand. So the work of strengthening our forces numerically must go on apace, gaining in momentum as each new recruit to the cause comes in. Side by side with this propaganda, in which the local centres are called to take a leading part, the constructive work done by Committee and Council must be steadily pursued. The extent of the ground to be covered is immense. The abilities of all who guide the helm will be taxed to the uttermost. But we look for slow consolidation, the germ of a fruitful co-operation among hospitals, small and large, and the beginnings of important reforms. When the College meets in 1919 (perhaps, who can say? in its own stately premises) it should be well on its way to endowment with a capital sufficient for present and future needs.

